# Laparoscopic Partial Nephrectomy for Renal-Cell Carcinoma During Pregnancy

**DOI:** 10.1089/cren.2015.0012

**Published:** 2016-02-01

**Authors:** Murat Binbay, Emrah Yuruk, Burak Ucpinar, Zerrin Binbay, Aykut Colakerol, Ahmet Yaser Muslumanoglu

**Affiliations:** ^1^ Department of Urology, Haseki Training and Research Hospital, Istanbul, Turkey.; ^2^ Department of Urology, Bagcilar Training and Research Hospital, Istanbul, Turkey.; ^3^ Department of Psychiatry, Haseki Training and Research Hospital, Istanbul, Turkey.

## Abstract

***Background:*** The incidence of renal-cell carcinoma (RCC) is low during pregnancy. There are different approaches for timing of surgery and treatment modalities for RCC in pregnant women in the literature. To our knowledge, this is the first laparoscopic partial nephrectomy case in a pregnant woman.

***Case Presentation:*** Herein, we present a 34-year-old woman with a renal mass at her 14th gestational week. She was admitted to our clinic after a right renal mass was incidentally diagnosed during routine antenatal ultrasonography. MRI revealed a completely endophytic tumor of 6 × 6.5 × 6.5 cm, located in the upper half of the right kidney. We performed laparoscopic partial nephrectomy in our patient and the postoperative course was uneventful.

***Conclusion:*** This is the first presented laparoscopic partial nephrectomy case in a pregnant patient. Nephron-sparing surgeries can be performed laparoscopically in appropriate sized renal tumors in suitable pregnant patients.

## Introduction and Background

The incidence of renal-cell carcinoma (RCC) is low among young people and even lower during pregnancy. When it is diagnosed in a pregnant woman, treatment decision and timing of the surgery become of utmost importance for survival of not only the mother but also the fetus. Surgical approaches to renal tumors have dramatically changed during the past decade. According to recent guidelines on renal tumors, partial nephrectomy has become the treatment of choice for T1 renal cancers.^1^ Moreover, as a result of the improvements in laparoscopic experience and technological advances in the surgical equipment, most of the partial nephrectomies are being performed by using minimal invasive techniques at centers of excellence.

Based on the current evidence, it is suggested that localized renal cancers are best treated by partial nephrectomy, if resection of cancer is technically feasible. In contrast, all pregnant women with renal cancer were treated by radical nephrectomy through either laparoscopic surgery or open surgery. We advocate that pregnant women with renal tumors are better to be treated by partial nephrectomy as minimally as possible, depending on surgeon's experience. In addition, timing of operation is crucial for RCC in pregnant women. Delaying the surgery to after birth may lead to lethal consequences if RCC is diagnosed in the early phase of pregnancy. Renshaw and colleagues mentioned that RCC in younger patients tends to be more aggressive than in older adults.^2^ Human embryo or fetus is most vulnerable to drugs and teratogen factors during the first trimester. If RCC is diagnosed during the third trimester, delaying the operation to after birth is a viable option, because of high risk of miscarriage.^3^ Therefore, operating a pregnant patient during the second trimester is preferable. However, there are certain articles that recommend surgery during the first trimester, even though it carries a slightly increased risk of miscarriage.^4^

To the best of our knowledge, we hereby report the first laparoscopic partial nephrectomy case during pregnancy.

## Clinical History

A 34-year-old pregnant lady at her 14th gestational week was referred to our clinic with an incidentally detected right renal mass during routine antenatal ultrasonography. She did not have any associated diseases and she was a nonsmoker. This was her first pregnancy. She did not have any family history of RCC and no genetic abnormalities were identified.

## Physical Examination and Diagnosis

**FIG. 1.**
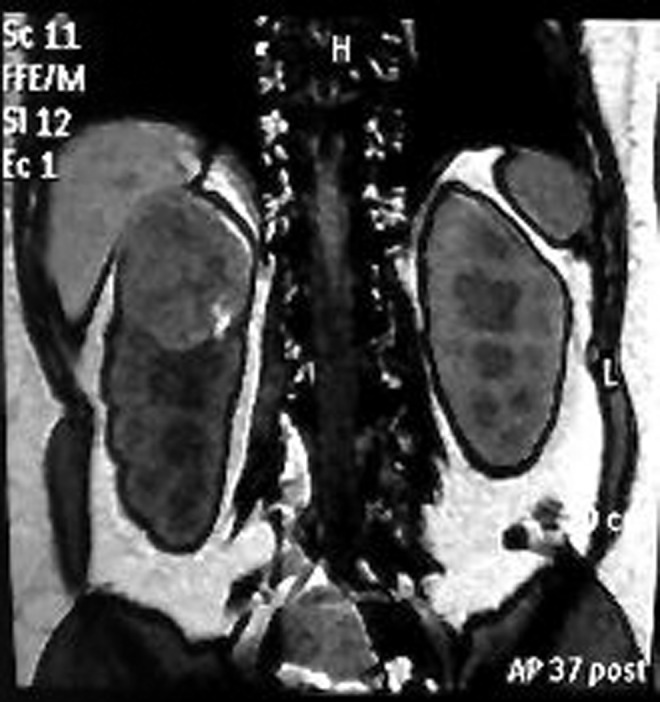
Preoperative MRI of the patient.

**FIG. 2.**
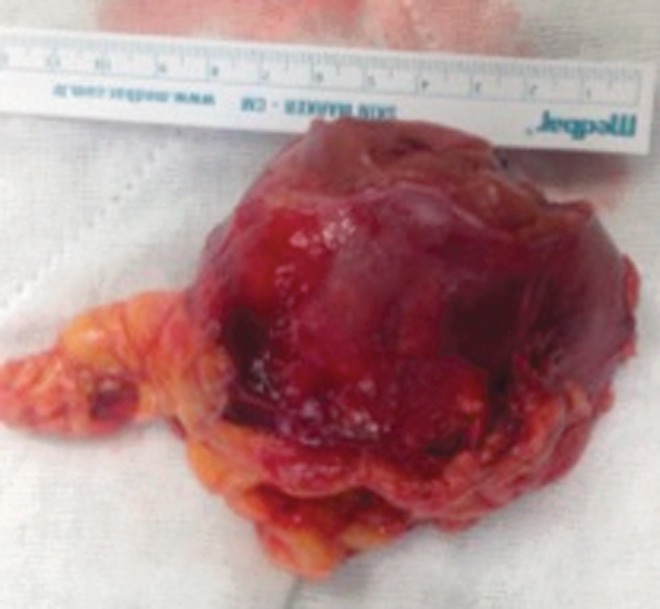
Macroscopic view of the resected tumor tissue.

Physical examination of the patient was unremarkable despite her enlarged uterus because of her pregnancy. Subsequent MRI showed a completely endophytic tumor of 6 × 6.5 × 6.5 cm, located in the upper half of the right kidney (Fig. 1), which was interpreted radiologicaly for RCC. Renal vein invasion or lymph node involvement was not detected. Her preoperative chest radiograph was normal and her liver enzymes and calcium levels were all within normal limits. Despite her concerns about possible consequences of delaying treatment until completion of pregnancy, she was keen to give birth to her child. Obstetrics and Gynecology Department recommended removing the tumor with a technique as minimally invasive as possible during the second trimester of pregnancy. Possible treatment alternatives were discussed with the patient and she decided to undergo laparoscopic partial nephrectomy. Intervention: An experienced laparoscopic surgeon performed right laparoscopic partial nephrectomy through the transperitoneal approach during the 16th gestational week. The pressure of the pneumoperitoneum was 12 mm Hg during surgery. The first 10-mm trocar was placed on 3 cm medial to umbilicus by the Hasson technique. The remaining four trocars, including three 5-mm and one 10-mm trocars, were placed in an L-shaped configuration. Meticulous dissection of the renal hilum allowed the surgeon to ligate small arteries supplying the renal tumor. After clamping the renal artery with a Bulldog clamp, the tumor was resected with cold cut. Hemostatic closure of the tumor bed and repair of the pelvicaliceal system were achieved with a 3/0 self-retaining barbed suture (V-Loc). Early unclamping technique was preferred. Renography was completed with 2/0 gluconate suture after application of Floseal. The tumor was removed through the expanded camera port incision and put into an organ bag (Fig. 2). At the end of the operation, retrograde Double-J stent was placed. Operation time and warm ischemia time were 165 and 24 minutes, respectively. Estimated blood loss was 200 mL during the operation and our patient did not require blood transfusion. No complication occurred during or after the surgery. Obstetrics and Gynecology consultation confirmed the survival of the fetus just before and after the operation.

## Follow-Up and Outcomes

**FIG. 3.**
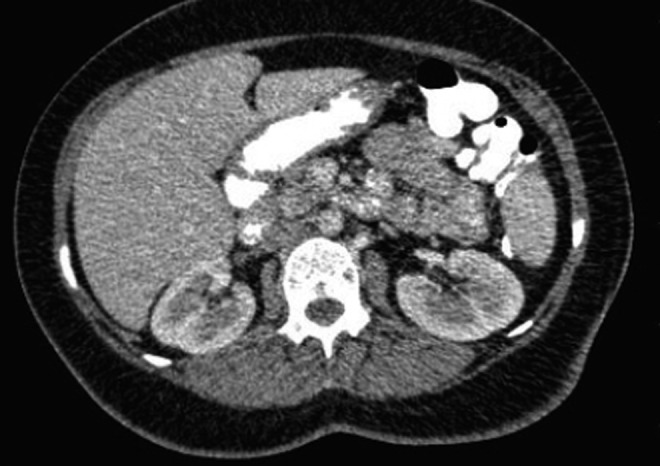
Postoperative CT scan of the patient.

The patient's presenting creatine level was 0.6 mg/dL. The estimated glomerular filtration rate (eGFR) decreased from 136 mL/minute/1.73 m^2^ to 119 mL/minute/1.73 m^2^ on the second postoperative day. The patient's GFR was 96 mL/minute/1.73 m^2^ on the 18th postoperative month during follow-up. The urethral catheter and drainage tube were removed on postoperative days 2 and 3, respectively. The patient was discharged from the hospital on the fourth day after surgery, uneventfully. The pathology report revealed clear RCC, Fuhrman grade 3 with negative surgical margins. According to the TNM classification system, it is classified as pT1b. During the 36th gestational week, a healthy male infant was born by cesarean section. We have followed our patient routinely after surgery. Thorax and abdominal CT scan during 9 and 18 months after the operation showed neither metastasis nor local recurrence (Fig. 3). We are in the 22nd postoperative month now. The patient's baby is perfectly normal and does not have any developmental birth defects. Although the baby is perfectly normal, routine follow-up will be performed to detect further cognitive or developmental defects.

## Abbreviations Used

CT: computed tomography
eGFR: estimated glomerular filtration rate
MRI: magnetic resonance imaging
RCC: renal-cell carcinoma
